# Future Time Perspective in Occupational Teams: Do Older Workers Prefer More Familiar Teams?

**DOI:** 10.3389/fpsyg.2017.01639

**Published:** 2017-09-26

**Authors:** Laura U. A. Gärtner, Guido Hertel

**Affiliations:** Department of Organizational and Business Psychology, Institute of Psychology, University of Münster, Münster, Germany

**Keywords:** future time perspective, age-related differences, older workers, teamwork, team preference, socioemotional selectivity theory

## Abstract

Working in teams is quite popular across different industries and cultures. While some of these teams exist for longer time periods, other teams collaborate only for short periods and members switch into new teams after goals are accomplished. However, workers’ preferences for joining a new team might vary in different ways. Based on Carstensen’s socioemotional selectivity theory, we predict that emotionally meaningful teams are prioritized when occupational future time perspective (OFTP) is perceived as limited. Building and expanding on studies outside of the work context, we expected that older as compared to younger workers prefer more familiar teams, and that this effect is mediated by workers’ OFTP. Moreover, we assumed that experimentally manipulated OFTP can change such team preferences. The hypotheses were tested in an online scenario study using three experimental conditions (within-person design). Four hundred and fifty-four workers (57% female, age *M* = 45.98, *SD* = 11.46) were asked to choose between a familiar and a new team in three consecutive trials: under an unspecified OFTP (baseline), under an expanded OFTP (amendment of retirement age), and under a restricted OFTP (insolvency of the current company). Whereas the baseline condition was always first, the order of the second and third conditions was randomized among participants. In the baseline condition, results showed the expected mediation effect of workers’ OFTP on the relation between workers’ age and preference for a familiar over a new team. Higher age was associated with more limited OFTP, which in turn was associated with higher preference for a familiar over a new team. Moreover, experimentally restricting OFTP increased preference for a familiar team over a new team regardless of workers’ age, providing further evidence for the assumed causal processes and showing interesting avenues for practical interventions in occupational teams.

## Introduction

Today, working in occupational teams is ubiquitous (Kozlowski and Ilgen, 2006; Mathieu et al., 2017). However, workers often change their team over the course of their careers, for instance, after a project cycle is concluded. While workers are often assigned to a team by the management, workers can also volunteer for or select a specific team. Regardless of such opportunities, workers have preferences about whom they want to work with, for instance, as a function of familiarity of the other team members (e.g., Glaman et al., 2002; Tett and Murphy, 2002; Bandiera et al., 2005). While some workers embrace new teams as learning opportunities and expansions of their occupational network, others might dislike such changes and rather prefer familiar teams. In the current research, we postulated that workers’ chronological age is a central influence on such team preferences, and that these age effects are mediated by workers’ occupational future time perspective (OFTP).

Our postulation is based on the socioemotional selectivity theory (e.g., Carstensen, 2006), which explains age-related changes in social behavior as a function of individuals’ perceptions of remaining time (Carstensen, 2006; Cate and John, 2007). A general assumption of this theory is that information acquisition and expansion of social networks are more strongly emphasized when future time is perceived as rather expansive and open-ended, whereas emotional well-being and the maintenance of existing social contacts increase in their relative importance when future time becomes perceived as limited (e.g., Carstensen, 2006). Indeed, empirical studies have shown that older persons generally prefer emotionally close and familiar over novel social partners (e.g., Fredrickson and Carstensen, 1990; Fung et al., 1999, 2001; Fung and Carstensen, 2006).

However, the central mechanisms described in the socioemotional selectivity theory are not assumed to be restricted to persons’ general lifespan but can be applied to different time spans (Carstensen et al., 2003). We applied the age-related dynamics described by the socioemotional selectivity theory to the context of working teams, and examined consequences of age-related changes in OFTPs. In the work context, occupational time refers to the time between entry and exit of individuals’ occupational activities. Therefore, OFTPs describe individuals’ perceived remaining time for their occupational activities (see Zacher and Frese, 2009, 2011). Please note that OFTP can refer to different occupational activities and related time spans depending on the focused frame of reference. For instance, occupational time more generally refers to the complete time span between starting a first career until retirement. However, organizational time refers to the time span between entering and leaving a specific work organization. Although the different focused frames of reference might sometimes lead to different future time perspectives that can even interact with each other (e.g., a relatively young worker having a long general occupational time perspective, but a limited organizational future time perspective due to a temporary work contract; e.g., Husman et al., 2016), we postulated that the direction of OFTP effects on the preference for familiar over new teams is similar regardless of the specific time span.

We assumed that OFTPs explain age-related differences in preferences for familiar over new teams. We measured age-related differences of OFTP as mediator between age and team-related preference. In addition to that we also manipulated participants’ OFTP in order to demonstrate its causal influence on participants’ preference of familiar over new teams (e.g., MacKinnon and Pirlott, 2014; Preacher, 2015) using a dynamic within-person design (see also Goldstein et al., 1994; Quené and van den Bergh, 2004). Each participant indicated their preferences for familiar or new teams in three conditions, i.e., during a first baseline condition and during two consecutive conditions with a temporary manipulation of OFTP. The temporary manipulations of OFTP were realized with a scenario approach including either an expansion of OFTP (amendment of retirement age by 10 years) or a limitation of OFTP (termination of employment due to insolvency of the current company). Due to the multilevel nature of our data, we could examine rather stable age-related differences in team-related preference as a function of workers general perceptions of OFTP (between-subjects effect), as well as temporary changes of team-related preferences in response to the experimental manipulation of OFTP (within-subjects effects; see multilevel logistic modeling, Van der Leeden, 1998; Quené and van den Bergh, 2004).

Our study contributes to existing literature in three ways. First, we tested the relationship between age and preference for familiar over new teams, adapting implications of socioemotional selectivity theory to occupational team settings. Second, we tested OFTP as mediating mechanism between age and preference for familiar over new teams using both correlational and experimental evidence (third contribution). Understanding such mediating processes not only contributes to the epistemic understanding and theory development, but also supports the development of practical interventions. In particular, documenting the central role of OFTP as effective mediator between workers’ age and team-related preference provide fruitful suggestions for HR management and companies to address challenges related to the increasing demographic changes in most countries worldwide (e.g., Hertel and Zacher, in press).

### Age, Occupational Future Time Perspective, and Team Preference at Work

Socioemotional selectivity theory maintains that the perception of time influences social goal-directed behavior and motivation (Carstensen, 1995, 2006). Depending on the temporal context, individuals set goals and adjust their motivational investment to reach these goals. In particular, the relative priority of specific goals might change as a function of individuals’ perceptions how much time is remaining. Hence, when time is perceived as limited – for instance, when people get older – attention shifts from expansive or “future-oriented goals to emotionally meaningful goals” (Carstensen et al., 2003; Fung and Carstensen, 2006, p. 248–249). As a result, the perceived boundaries on time are assumed to affect social motivation and goal orientation in the way that the regulation of emotional states becomes prioritized. By spending time with emotionally meaningful partners, the individual’s benefit is in the contact itself because positive feelings arise out of this social contact (i.e., effective emotion regulation; Carstensen et al., 2003). In contrast, when pursuing expansive or open-ended goals, individual focus more on potential long-term payoffs, such as gathering knowledge or future career opportunities.

Furthermore, the perceived remaining time is also assumed to affect individuals’ preference for familiar or unknown interaction partners. Prior research has shown that individuals with a limited perceived future time maximize contact with familiar or emotionally close partners and minimize interaction with unknown or less familiar persons so that they can conserve energy and regulate their affect (Carstensen et al., 1999; Fung et al., 1999). By focusing their resource usage on familiar partners, persons assimilate to the perceived shrinking time horizon. Such motivational shifts can be related to persons’ lifespan in general, but are also possible in more specific life domains or contexts, such as, for instance, leisure or work (see also Peetsma and van der Veen, 2011).

Upon entering a career, individuals are usually inexperienced and need to learn new skills and expertise through gathering information and socializing. In this case, the perceived time perspective for this new activity is rather wide and open-ended. Zacher and Frese (2009, 2011) adapted the general assumptions of socioemotional selectivity theory to employees’ perceptions of remaining time and opportunities at work, and showed that age and OFTP were negatively related (see also Weikamp and Göritz, 2015). Moreover, various researchers have found that persons’ work values differ as a function of their age, with younger workers placing higher values on information gathering and career orientation whereas older workers being more likely to prefer emotionally meaningful goals and generativity motives at work (e.g., Ng and Feldman, 2010; Kooij et al., 2011; Hertel et al., 2013; Hommelhoff et al., 2017). Furthermore, an expanded time perspective seems to facilitate social networking activities and contact frequency at work (e.g., Wrzus et al., 2013). Thus, at the beginning of a professional career, workers seem to focus more on gathering new information and knowledge. However, toward the end of a career, workers more strongly try to save resources by avoiding negative emotions (Hertel et al., 2013) and pursuing activities that increase experiences of meaningfulness, such as supporting other colleagues (Ng and Law, 2014).

Based on the socioemotional selectivity theory, we assumed that workers’ age influences their preference for familiar over new teams. Joining or staying in a more familiar team implies that work conditions and interaction partners are more predictable and reliable, and that workers’ positive affect might arise out of the social contact itself. In contrast, joining a new team might come with more unknown consequences and therefore require unpredictable amounts of resources to handle upcoming tasks or struggles. However, a new team also includes learning opportunities and potential expansions of the occupational network. Thus, we hypothesized that older as compared to younger workers are more likely to favor familiar teams over new teams because familiar teams provide more opportunities for resource conservation and emotion regulation. Moreover, we proposed that OFTP mediates the link between age and preference for familiar over new teams. More formally, we postulated:

H1: Workers’ chronological age is positively correlated with their preference for familiar over new teams.
H2: The relationship between workers’ age and their preference for familiar over new teams is mediated by their occupational future time perspective.

In addition, we also assumed that workers’ preference for familiar over new teams (and vice versa) can be affected by *contextual* changes of their OFTP in addition to rather stable age effects. Such contextual changes can be caused by unforeseen accidents or deadlines, economic shortfalls, or legislative changes such as changes in retirement age. Moreover, contextual effects on OFTP can also refer to more specific occupational activities, such as working in a specific company (e.g., Husman et al., 2016). In general, an open-ended OFTP should lead to relatively high preference for new teams over familiar teams because this future-oriented goals are more important, and new teams provide additional new contacts and learning opportunities (Fredrickson and Carstensen, 1990; Carstensen, 2006). However, when workers’ OFTP is restricted their preference for new over familiar teams should be rather low, instead, emotionally meaningful relationships should be more important to regulate affect, optimize the usage of resources, and compensate for losses (Baltes and Carstensen, 1996; Carstensen, 2006).

Existing research has shown that future time perspective can change behavior and motivation in different contexts, such as individuals’ lifestyle choices (Tasdemir-Ozdes et al., 2016), job-crafting intentions (Kooij et al., 2016), goal setting and tracking (Ko et al., 2014), and choice of social partners (Fung et al., 1999; Hommelhoff et al., 2017). However, there is no empirical evidence so far that contextual changes in OFTP can also change team-related preferences at work. Examining potential effects of such contextual changes of OFTP provide interesting insights for potential practical interventions in work organizations, for instance, in order to motivate older workers to join new teams. Moreover, examining contextual changes of OFTP in a controlled experimental design contributes a more conservative test of the causal influence of OFTP on preferences for familiar over new teams, which is inherent in the assumed mediation process specified in Hypothesis 2.

More formally, we assumed that temporary changes in OFTP have a causal influence on team-related preference. Specifically, we hypothesized that contextual influences can temporarily affect persons’ preference for familiar over new teams at work:

H3: Temporary changes of occupational future time perspective causally affect workers’ preferences for familiar over new teams, insofar that expansions of occupational future time perspective decrease, and limitations of occupational future time perspective increase workers’ preferences for familiar over new teams.

## Materials and Methods

### Participants

The study was conducted using a German online panel (a pool of registered persons who have agreed to take part in web-based studies) on psychological research^1^. All panel members who matched the two criteria of the panel filter “working” and “between 18 and 67 years old”^2^ were invited by email to voluntarily participate. Six hundred and three participants followed the link to the questionnaire, and 454 participants (drop-out rate 25%) could be included in the analyses. The excluded participants (25%) did not differ demographically from the included participants. The sample consisted of 57% females, 42% males, and 1% not specified. Workers were between 18 and 67 years of age (*M* = 46 years, *SD* = 11.46) and had worked for about 12 years (*SD* = 10.77) for their present company. In our study more than 60% of the participants did social or entrepreneurial work (based on Holland’s RIASEC model, 1997) and had a university degree. The participants estimated that on average about 51% of their daily work was teamwork, and 59% of participants preferred teamwork over working alone. In addition, the perceived physical health was described as rather good (*M* = 3.94, *SD* = 0.87).

### Procedure

The study has a mixed 3 (contextual constraints: baseline, extended, or limited OFTP) × 2 (sequential order of extended and limited OFTP conditions) design. The three conditions were nested within each participant with the order of the conditions differing between participants. For testing Hypotheses 1 and 2, we considered only the baseline condition. To examine Hypothesis 3, we used the experimental within-person design to show that temporary changes in OFTP can change participants’ preference of familiar over new teams. Furthermore, we accounted for differences on the between-group level such as age and stable OFTP (between-group design). In doing so, we were able to explore changes in participants’ team-related preferences triggered by the interaction of experimentally manipulated (temporary) future time perspective and more stable age-related differences between workers.

At the beginning of the survey, participants were given instructions and were assured that all data would be handled in an anonymized form and only used for scientific purposes. All participants started with the unspecified baseline condition. No specific instructions were given for the baseline condition; participants were simply asked which team they would prefer:

Imagine you are working together in a team. Due to reorganization at your company, you have the possibility to change teams or remain in your existing team. Assuming that the two following teams are available, which team would you choose?

Afterward, participants had to select one out of two teams. The familiar team was described as *Team A: existing/known team, all tasks and responsibilities are clear, colleagues are well-known, knowledge and expertise are established.* The new team was described as *Team B: new team, tasks seem to include the opportunity to learn something new, colleagues are unknown, could provide future career opportunities*.

After participants had indicated the preferred team, the second team preference task followed, this time depending on the experimental order condition. In the extended OFTP condition, participants read:

*Now imagine the following situation: Last week you were informed by your management that the government has adopted a law which increases the mandatory retirement age by 10 years. In addition, occupational health protections at work have been improved such that it is possible for all to work longer in good physical condition*.

In the limited OFTP condition, participants read:

Now imagine the following situation: You were told by your management that your company is in the red again this year and is bankrupt. The company’s continued existence is ruled out. One year remains for all employees until lay-off; however, there is still enough work so that all employees can continue their work until the end of the year.

After each scenario, participants were asked again which team they would prefer with the same description of the two teams in all three scenarios. The assessment of stable OFTP was conducted after the three team preference measures, together with the demographic variables. We decided to measure all time-related variables after the experimental conditions because we were concerned about potential demand (OFTP) and self-stereotyping effects (e.g., Desmette and Gaillard, 2008; Posthuma and Campion, 2009; Meisner, 2012). In addition, stable OFTP was considered to be highly related to participants’ age, which is an objective variable making recursive effects of the dependent variable on age unlikely. Please note that we measured OFTP with respect to participants’ real occupational life, not with respect to the imagined future time scenarios that were part of the experimental manipulation^3^. Thus, we assumed that participants were able to imagine an expanded or limited occupational future time scenario and anticipate preferences based on those without changes in their general OFTP with regard to their real occupational life (see also Fredrickson and Carstensen, 1990; Fung et al., 1999, for similar assumptions).

At the end of the survey, participants were offered feedback on their personal OFTP score. This feedback was provided anonymously by the system using benchmarks derived from a previous unpublished study.^4^ Furthermore, this research was part of a larger research project.^5^ For the current study, we focused on perceived OFTP and demographics such as age, gender, organizational as well as job tenure, percentage of teamwork, organizational support, attitude toward teamwork in general, education, and physical health.

This study adheres to the recommendations of the Federation of the German Psychologists Association’s Code of Ethics. The online panel platform used for this research^6^ is a joint project of four German universities, and complies with the scientific standards in psychology research. Approval was given by the project manager dedicated to psychology. All subjects participated voluntarily in the survey in accordance with the Declaration of Helsinki. No ethical review or approval was required for this study under the national or international requirements.

### Measures

To develop the experimental scenarios, we adopted existing scenarios from research on social partner selection (Fredrickson and Carstensen, 1990; Fung et al., 1999). In their studies, Fung et al. (1999) used a baseline condition, an extended future time condition (i.e., participants were informed that they will live 20 years longer than expected), and a limited future time condition (i.e., emigration to another country in the next weeks). As expected by the authors, participants were less likely to select known social partners in the extended future time condition. In the limited future time condition, the participants preferred spending time with familiar partners. The authors compared the participants’ preference for the familiar social partners in the contextually changed scenarios with the baseline condition. This research showed that the manipulation of future time perspectives can significantly affect participants’ preferences for social partners (Fredrickson and Carstensen, 1990; Fung et al., 1999). Building on and extending this research, we adapted the scenarios to the context of occupational teamwork. In doing so, we operationalized the contextual extension of OFTP with respect to participants’ more general occupational future time (amendment of retirement age by 10 years) whereas the contextual restriction of OFTP was realized with respect to participants’ organizational future time (termination of employment due to insolvency of the current company) as a more specific aspect of OFTP. However, the postulated direction of OFTP on the preference for familiar over new teams was assumed to be the same regardless of the specific frame of reference of OFTP.

The participants’ team-related preferences were coded “1” for the familiar and “0” for the new team. Order of the contextual variations of OFTP were coded “0” when the extended OFTP condition preceded the limited OFTP condition, and “1” when the limited OFTP condition preceded the extended OFTP condition.

Participants’ stable OFTP was measured with respect to their real occupational life using items from Zacher and Frese (2009, 2011; Zacher, 2013) based on Carstensen and Lang’s (1996) German future time perspective scale. We followed Zacher’s (2013) suggestion to include only 8 of the 10 items matching to three independent latent variables. Factor analyses suggested that the items of the OFTP scale load on three subscales: the perceived Remaining Time, the Focus on Opportunities, and the Focus on Limitations before leaving the workforce (Zacher, 2013). We included these subscales in our analyses because they might influence participants’ preference for familiar over new teams in different ways. Our experiment built on temporary changes in remaining occupational future time, therefore the subscale Remaining Time (measured with three items, for instance, *“Most of my occupational life lies ahead of me.*” Cronbach’s α = 0.80; see also Zacher and Frese, 2009; Zacher, 2013) might be particularly relevant in the relation between age and participants’ team-related preference. The other two subscales Focus on Opportunities (measured with three items, for instance, *“My occupational future is filled with possibilities.”* Cronbach’s α = 0.90), and Focus on Limitations (measured with two items, for instance, *“As I get older, I begin to experience time in my occupational future as limited.”* Intercorrelation = 0.69, *p* < 0.001; see also Cate and John, 2007; Zacher, 2013) might have lower but still significant effects in this regard. Furthermore, we also included the item: *“I could do anything I want in my occupational future.”* Zacher and Frese (2011) used four items to measure the Focus on Opportunities scale and Zacher (2013) showed a moderate factor loading for this item. Therefore, we included this item in the overall OFTP scale (Cronbach’s α = 0.91). Participants answered on a Likert scale ranging from *“does not apply at all”* (1) to *“applies completely”* (7) (Zacher and Frese, 2009).

Moreover, subjective health was considered as a control variable. Prior studies have shown positive correlations between OFTP and subjective health (Zacher and Frese, 2009; Kooij et al., 2013; Hoppmann et al., 2015). We assessed physical health with one item: *“How would you describe your state of health in general?”* on a five-point Likert scale ranging from very bad (1) to very good (5).

The following variables were measured at the end of the study: participants’ age, organizational and job tenure, gender, highest educational qualification, percentage of their teamwork in their current job (from 0 to 100%), attitude toward teamwork in general, perceived organizational support, and work environment (adapted from Holland, 1997).

### Analytical Procedure

To test the main effects of participants’ age on their preference for familiar over new teams (H1) and the assumed mediation of OFTP (H2), we conducted multiple logistic regression analyses. For OFTP, we calculated an average scale score with higher OFTP scores indicating more open-ended future time perspectives, and lower OFTP scores indicating more limited future time perspectives. Furthermore, to compute the indirect effect of age on participants’ preference for familiar over new teams (mediated by OFTP), we used the PROCESS macro for SPSS by Hayes (2013) including 5,000 bootstrapping samples. Bootstrapping contains random resampling with replacement. IBM SPSS Statistics 24 was used for the analyses. Additionally, we calculated multiple logistic regression analyses for the mediating role of the three subscales of OFTP on the relation between age and participants’ preference for familiar over new teams. The three subscales were designated as Remaining Time (high scores indicate perceived long future time remaining), Focus on Opportunities (high scores indicate plenty perceived future opportunities), and Focus on Limitations (high scores indicate perceived few future limitations).

To accommodate the multilevel nature of our study and the nested structure of our data (team preference decisions nested within each participant), we used multilevel path modeling. In doing so, we followed Quené and van den Bergh’s (2004) suggesting a multilevel random coefficients model instead of calculating an ANOVA with repeated measures. First, the multilevel approach allows for handling unbalanced data and does not require sphericity. Second, a multilevel approach takes the hierarchical data structure (design effect) into account and considers intra-class correlation (Goldstein et al., 1994). Third, all participants could be included regardless of missing data points, maintaining the planned power of the experimental design (Quené and van den Bergh, 2004).

To analyze the influence of the temporary limitation or expansion of OFTP, we added two dummy variables: one for the limited and one for the extended OFTP condition. These dummies were used as independent variables influencing participants’ preference for familiar over new teams as binary dependent variable on the within level (Snijders and Bosker, 2012). We considered the dichotomous nature of the dependent variable by using multilevel logistic regression analysis. We used MPLUS 7.4 (Muthén and Muthén, 2012, Los Angeles, CA, United States) for the two-level analysis with maximum likelihood estimation. In addition, we centered the between-factor variables age and OFTP (covariates) around the grand mean (Hofmann and Gavin, 1998). Order of experimental conditions was entered at the between level of analysis.

## Results

### Descriptive Statistics

Means, SD, and intercorrelations of main variables are displayed in **Table 1**. As expected, participants’ age and OFTP were negatively correlated (*r* = -0.65). However, participants’ age was unrelated to preferences for familiar over new teams in all three experimental scenarios, which is inconsistent with Hypothesis 1. In contrast, participants’ stable OFTP was negatively related with the preference for familiar over new teams in all three experimental conditions (baseline condition: *r* = -0.15, extended OFTP condition: *r* = -0.17, and limited OFTP condition: *r* = -0.12), which is partly consistent with Hypothesis 2 and our expectations that a low OFTP is associated with rather low preferences for new teams. The correlation in the limited OFTP condition was slightly lower than in the other two conditions but the difference was not significant.^7^ Order of conditions was negatively correlated with participants’ preference for familiar over new teams in the limited OFTP condition (*r* = -0.11). In this condition, the order condition “baseline – expansion – limitation” lead to a stronger preference for the familiar team as compared to “baseline – limitation – expansion” condition. Moreover, perceived health was correlated with age (*r* = -0.12), OFTP (*r* = 0.27), and team preferences in the baseline (*r* = -0.12), extended OFTP (*r* = -0.12), and limited OFTP (*r* = -0.06) conditions. Based on these correlations, we considered the order of conditions and perceived health as control variables in our analyses. Similar to prior studies (e.g., Fredrickson and Carstensen, 1990), no gender differences were observed with respect to the main dependent variable.

**Table 1:** Descriptive statistics and correlation among variables.

Variables	*M*	*SD*	1	2	3	4	5	5.1	5.2	5.3	6	7	8	9	10	11
(1) Team preference baseline	0.46	0.50	–													
(2) Team preference expansion	0.49	0.50	0.62**	–												
(3) Team preference limitation	0.62	0.49	0.16**	0.18**	–											
(4) Age	45.98	11.46	0.03	0.03	0.02	–										
(5) OFTP	3.96	1.43	-0.15**	-0.17**	-0.12*	-0.65**	–									
(5.1) Remaining time	3.48	1.64	-0.11*	-0.13**	-0.12*	-0.74**	0.91**	–								
(5.2) Focus on opportunities	4.38	1.62	-0.20**	-0.22**	-0.12*	-0.44**	0.89**	0.73**	–							
(5.3) Focus on limitations_recode_	4.02	1.81	-0.01	-0.02	-0.08	-0.56**	0.79**	0.65**	0.53**	–						
(6) Gender	1.59	0.51	0.06	0.080	0.02	-0.03	-0.02	0.00	-0.06	0.02	–					
(7) Education	–	–	-0.06	-0.08	0.02	-0.05	0.11*	0.10*	0.12**	0.03	-0.08	–				
(8) Subjective health	3.94	0.87	-0.12*	-0.12**	-0.06	-0.12*	0.27**	0.20**	0.27**	0.22**	-0.05	0.20**	–			
(9) Organizational tenure	11.68	10.77	0.14**	0.07	0.05	0.53**	-0.43**	-0.42**	-0.36**	-0.34**	0.02	-0.11*	-0.06	–		
(10) Job tenure	16.07	12.45	0.08	0.07	-0.02	0.58**	-0.41**	-0.44**	-0.31**	-0.34**	-0.02	-0.06	-0.14**	0.60**	–	
(11) Percentage of teamwork	50.50	29.46	0.06	0.01	0.00	-0.02	0.11*	0.07	0.16**	0.07	-0.02	-0.04	0.02	0.01	0.04	–
(12) Order of conditions	–	–	0.03	-0.06	-0.11*	0.05	-0.01	-0.02	-0.01	-0.02	0.01	0.05	0.05	0.03	0.00	-0.06

*N = 454. Team preference 0 = new team, 1 = familiar team; and baseline, expansion, and limitation specifies the experimental conditions; occupational future time perspective (OFTP) and its three subscales Remaining Time (RT), Focus on Opportunities, Focus on Limitations_*recode*_ 1 = limited future time perspective, up to 7 = open-ended future time perspective; gender 1 = male, 2 = female, 3 = not specified; education 1 = without graduation, up to 4 = university degree; % of teamwork in their current job; order of conditions 0 = baseline, expansion, limitation and 1 = baseline, limitation, expansion*.

^∗^*p < 0.05. ^∗∗^*p* < 0.01*.

Furthermore, we found moderate correlations on the within-person level for the three team preferences, ICC = 0.48 (Intra-class Correlation; see Quené and van den Bergh, 2004). This ICC score indicates dependency in the data, prescribing additional multilevel analyses in order to avoid an underestimation of the standard error and alpha error inflation (Cress, 2008; Huang, 2017).

### Testing Main and Mediation Effects of Age on Team Preference

Multiple logistic regression analyses of participants in the unspecified baseline condition showed no higher preference for the familiar over the new team as a function of participants’ age (Hypothesis 1), *b* = 0.01, Wald = 0.16, *p* = 0.66. Thus, Hypothesis 1 was rejected.

**FIGURE 1:**
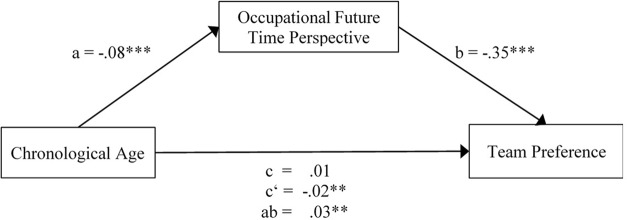
The tested model with effect sizes. The small letters describe the paths of the mediation analysis (c = total; c’ = direct; ab = indirect effect). For occupational future time perspective high scores indicate more open-ended future time perspectives, and lower scores more limited ones. Team preference is coded as “1” for the familiar and “0” for the new team. ^∗^*p* < 0.05, ^∗∗^*p* < 0.01, ^∗∗∗^*p* < 0.001, two-tailed.

However, results showed that age effects on participants’ preference for familiar over new teams were mediated by participants’ OFTP. The effects between age and OFTP (path a in **Figure 1**), *b* = -0.08, *t* = -17.66, *p* < 0.001, and OFTP and preference for familiar over new teams (path *b*), *b* = -0.35, *Z* = -3.61, *p <* 0.001, were significant. As evident in the test of H1, the total effect between age and preference for familiar over new teams was not significant. However, the total effect consists of the sum of the direct and indirect effect (e.g., MacKinnon et al., 2007; Hayes, 2013). Age was a significant predictor of participants’ preference for new over familiar teams after controlling for the mediator OFTP (direct path c’), *b* = -0.02, *Z* = -2.12, *p <* 0.05. If the relation between an independent and a dependent variable becomes larger by including a third variable in the analysis, a suppression effect becomes conceivable (Tzelgov and Henik, 1991; MacKinnon et al., 2000). This implies that opposing signs of the direct and indirect effects could cancel each other out. Indeed, mediation can exist even if the overall relation between X and Y is non-significant (MacKinnon et al., 2000). In addition, the paths between age and OFTP, and between OFTP and team-related preference were significant when controlling for age. As a consequence, we performed a mediation analysis using bootstrapping with bias-corrected confidence estimates (Preacher and Hayes, 2004). We used the 95% bootstrap confidence interval based on 5,000 bootstrap samples to test if the indirect effect is different from zero (Preacher and Hayes, 2008). The results of the analysis showed that age indirectly affected participants’ preference for the familiar over the new team through the mediator OFTP (*b* = 0.03; CI = 0.01 to 0.04). The full analysis is shown in **Table 2** and **Figure 1** illustrates the assumed model with effect sizes. The sum of the negative direct (path c’ = -0.02) and positive indirect effect (path ab = 0.03) indicates the non-significant total effect (path c = 0.01). This result implies the existence of a suppressor effect or inconsistent mediation (MacKinnon et al., 2007). Together, Hypothesis 2 assuming that OFTP mediates the relation between age and participants’ preference for familiar over new teams was supported.

**Table 2:** Mediation analysis of the mediating role of OFTP on the relation between age and participants’ (team) preference for familiar over new teams in the baseline condition (between subjects).

Path	Effect	Criterion	Predictor	Coefficient	*SE*
C	YX	Team preference	Age	0.01	0.10
B	YM.X	Team preference	OFTP	-0.35^∗∗∗^	0.14
c’	YX.M	Team preference	Age	-0.02^∗^	0.13
A	MX	OFTP	Age	-0.08^∗∗∗^	0.04
	Control	OFTP	Health	0.32^∗∗∗^	
	Control	Team preference	Health	-0.17	

*N = 442. Criterion = Y, team preference in the baseline condition and coded as “1” for the familiar and “0” for the new team. M = mediator OFTP. X = predictor chronological age. Control = control variable subjective health (health)*.

^∗^*p < 0.05, ^∗∗^*p* < 0.01, ^∗∗∗^*p* < 0.001, two-tailed*.

Additionally, we also examined the mediation effects of the three OFTP subscales for exploratory reasons. We found a mediation effect for the subscale Remaining Time, with a result pattern similar to the overall mediation effect of OFTP (i.e., suppressor effect, MacKinnon et al., 2000). The effect between age and Remaining Time (path a), *b* = -0.10, *t* = -23.13, *p<*0.001, and Remaining Time and preference for familiar over new teams (path b), *b* = -0.30, *Z* = -3.18, *p <* 0.001, were significant. Similar to the OFTP mediation analysis, age was a significant predictor of participants’ preference for new over familiar teams after controlling for the mediator Remaining Time (direct path c’), *b* = -0.03, *Z* = -2.16, *p <* 0.05. Furthermore, the bootstrapping analysis showed that age indirectly affected participants’ team-related preference through the mediator Remaining Time (*b* = 0.03; CI = 0.01 to 0.05). For the subscales Focus on Opportunities and Focus on Limitations, no mediating effect between age and preference for familiar over new teams was found.^8^ These results indicate that the subscale Remaining Time explained most parts of the relation between age and preference for familiar over new teams. The full analysis is shown in **Table 3**.

**Table 3:** Mediation analysis of the mediating role of RT on the relation between age and participants’ (team) preference for familiar over new teams in the baseline condition (between subjects).

Path	Effect	Criterion	Predictor	Coefficient	*SE*
C	YX	Team preference	Age	0.01	0.10
B	YM.X	Team preference	RT	-0.30***	0.09
c’	YX.M	Team preference	Age	-0.03*	0.01
A	MX	Remaining time	Age	-0.10***	0.01
	Control	Remaining time	Health	0.22***	
	Control	Team preference	Health	-0.22	

*N = 442. Criterion = Y, team preference in the baseline condition and coded as “1” for the familiar and “0” for the new team. M = mediator RT. X = predictor chronological age. Control = control variable subjective health (health)*.

^∗^*p < 0.05, ^∗∗^*p* < 0.01, ^∗∗∗^*p* < 0.001, two-tailed*.

### Effects of the Experimental Manipulation of OFTP

**FIGURE 2:**
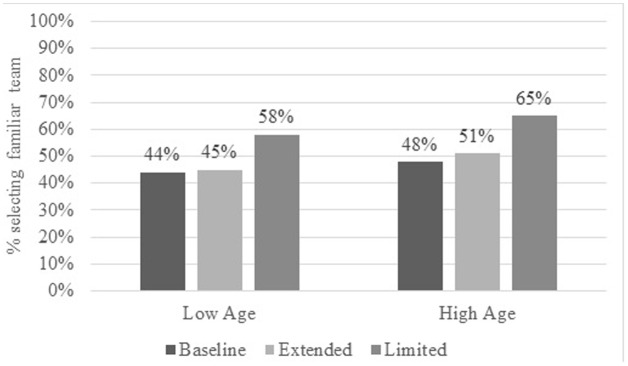
Participants’ preferences for the familiar team by age and (experimental) influences on occupational future time perspective (contextual scenarios: baseline, extended, and limited). The two age groups (low and high) were divided by median split at 47 years (n_old_ = 231, M_old_ = 54.86; SD_old_ = 4.88; n_young_ = 205, M_young_ = 35.95, SD_young_ = 7.77).

To test whether the contextual variation of (temporary) OFTP affected participants’ preferences for familiar over new teams, we used multilevel path modeling with MPLUS 7.4. The ICC score for the preference decisions suggested multilevel analyses to consider the influence of the contextual changes in momentary OFTP. We conducted a two-level analysis since each participant ran through all three conditions (within-subject ICC for the team preferences = 0.48). According to this, we added age, OFTP, and order of the conditions as between-level covariates to capture the effect of the experimental variation on the within-person level. The dependent variable was again participants’ preference for the familiar over the new team. The results showed that the contextual expansion of (temporary) OFTP had no influence on participants’ preference for familiar over new teams, *b* = 0.13 *p* = 0.43, OR = 0.88. However, the contextual limitation of (temporary) OFTP led to a higher preference for the familiar over the new team, *b* = 0.95, *p <* 0.001, OR = 0.39, even when controlling for participants’ stable OFTP scores, age, and order of conditions (see **Table 4**). Thus, Hypothesis 3 was partially confirmed by these data. The contextual limitation of temporary OFTP increased the preference for the familiar over the new team. This evidence supports the assumed causal influence of OFTP on preferences for familiar over new teams. However, we could not show that the contextual expansion of OFTP decreases participants’ preference for familiar over new teams. For illustration purposes, **Figure 2** shows the percentage of selections of the familiar team as a function of experimental manipulation and participants’ age (median split).

**Table 4:** Multilevel analysis of the effect from experimental expansion and limitation of temporary OFTP on participants’ (team) preferences for familiar over new teams (within level).

	Coefficient	*SE*	OR	*R*^2^
*Level 1 variables*				0.05^∗∗^
Contextual expansion	0.13	0.17	0.88	
Contextual limitation	0.95***	0.17	0.39	
*Level 2 variables*				0.13^∗∗^
Age	-0.03**	0.01		
OFTP	-0.52***	0.10		
Order of conditions	-0.52*	0.21		

*N = 442. Team preferences as the dependent variable included the team decisions of all three conditions nested within each participant and coded as “1” for the familiar and “0” for the new team. Contextual expansion = dummy variable for the extended OFTP condition. Contextual limitation = dummy variable for the limited OFTP condition. OFTP is occupational future time perspective. Order of conditions was coded as “0” = baseline, expansion, limitation (n_*order0*_ = 237) and “1” = baseline, limitation, expansion (n_*order1*_ = 205). The *R*^2^ in the logistic regression follows the underlying continuous latent response variable approach (see McKelvey and Zavoina, 1975)*.

^∗^*p < 0.05, ^∗∗^*p* < 0.01, ^∗∗∗^*p* < 0.001, two-tailed*.

## Discussion

This study investigated the influence of chronological age and OFTP on workers’ preference for familiar over new teams. Based on the socioemotional selectivity theory, we expected that older workers prioritize familiar over new teams because their OFTP is rather limited. Thus, we assumed that the effects of workers’ age are mediated by OFTP. Moreover, we examined the assumed mediating role of OFTP more rigorously by experimentally manipulating temporary OFTP through a contextual expansion and limitation. In addition to providing evidence for the assumed causal effect of OFTP on preferences for familiar over new teams, the examination whether temporary changes of OFTP can also affect team-related preferences might also provide interesting implications for practical interventions.

The hypotheses were examined with 454 workers recruited via an online panel and representing a broad variety of branches and company sizes. Interestingly, we found no total effect of age on workers’ preference for familiar over new teams. Instead, the mediation analyses revealed opposing direct and indirect effects of age on workers’ preference for familiar teams mediated by OFTP. These opposing effects seem to have counterbalanced each other’s influence, resulting in a non-significant total effect (see MacKinnon et al., 2007). In the baseline condition, older workers indicated a more limited OFTP, and a limited OFTP increased the preference for the familiar team, resulting in a positive indirect effect consistent with Hypothesis 2. However, OFTP could not explain the whole effect of age on team-related preference. Somewhat surprisingly, the observed direct effect of age on team-related preference suggested that older workers prioritized the new team over the familiar team more strongly than younger workers in our sample. This result is partly in conflict with Hypothesis 1, and with the assumptions of socioemotional selectivity theory (Carstensen, 2006). We also considered the causal influence of OFTP on participants’ preference for familiar over new teams using two different experimental scenarios. Contextually extending temporary OFTP had no effect on participants’ preference for familiar over new teams. However, contextually restricting participants temporary OFTP significantly increased the preference for familiar over new teams regardless of age and participants’ stable (general) OFTP score. The result for the contextual limitation of OFTP is consistent with our assumption based on socioemotional selectivity theory.

### Theoretical Implications

So far, existing research on age and teamwork focused mainly on age effects at the team level, such as age diversity in teams (e.g., Balkundi et al., 2007; Schneid et al., 2016). The results of the current study extend previous research on age effects at work on the individual level and shed light on the relation between age, preference for familiar over new teams, and the mediating effects of OFTP. Socioemotional selectivity theory predicts that people prioritize familiar interaction partners when remaining time is limited (Carstensen, 2006). However, the data of the current study revealed no direct age effects on participants’ preference for familiar teams. Interestingly, by including OFTP as control variable in the analysis, older workers were even more likely to prefer new over familiar teams (direct effect in the mediation analysis). These unexpected findings might suggest that variables other than OFTP are involved in the influence of age on preference for familiar over new teams. However, additional analyses of our data did not reveal mediating influences of health, education, tenure, or organizational support on the relation between age and preference for familiar teams. Moreover, exploratory analyses considering OFTP as moderator of the relation between age and team-related preference did not reveal any significant effect, *b* = -0.01, *Z* = -1.15, *p* = 0.25. Nevertheless, future research might consider other age-related process factors. Moreover, socioemotional selectivity theory and associated life-span research often consider ‘old age’ as 80 years and higher. In contrast, most workers retire from occupational life in their early 60s (OECD, 2010; He et al., 2016). Thus, retiring workers might be too ‘young’ to be regarded as ‘old’ for the expected age effect. This suggests the importance of assessing OFTP in addition to chronological age in work-related studies.

However, in accordance with socioemotional selectivity theory (Carstensen, 2006), our results demonstrated that older workers were more likely to prefer familiar over new teams when the effect was mediated by OFTP. By adding OFTP as a third variable, the relation between age and participants’ preference for familiar over new teams became larger. We found different signs for the direct and indirect effects of age on participants’ preference for familiar over new teams mediated by OFTP, suggesting a suppressor effect (i.e., inconsistent mediation; MacKinnon et al., 2007), which is rare but possible (MacKinnon et al., 2000). These results illustrate the importance of measuring (instead of just assuming) OFTP as a mediating process variable to better understand the relationship between age and participants’ team-related preference. Furthermore, in additional analyses we examined three subscales of OFTP: Remaining Time, Focus on Opportunities, and Focus on Limitations (Zacher, 2013). We found that the mediating influence of OFTP is best explained by the subscale Remaining Time, whereas no mediating effect of the other two subscales could be observed. In line with Carstensen’s (2006) socioemotional selectivity theory, this result might indicate that with higher age, workers’ perceived Remaining Time at work decreases and emotionally meaningful goals, such as familiar relationships, are prioritized. By selecting familiar teams, resources might be better optimized and losses compensated because the potential work situation is already known (see also Baltes and Carstensen, 1996). However, preference for familiar or new teams might additionally be influenced by the job itself or the industry involved. For example, a physically demanding job might decrease workers’ health more strongly, with related effects on workers’ OFTP. As a result, early retirement might be more likely (e.g., Blekesaune and Solem, 2005) and working with familiar teams might be preferred.

Finally, our study tested the causal effect of OFTP on preferences for familiar over new teams via two experimental variations of temporary OFTP. The results showed that the contextual expansion of temporary OFTP had no effect on participants’ preference for familiar over new teams. However, when OFTP was temporarily restricted, workers – regardless of their age – more often preferred the familiar team. The result for the contextual limitation of temporary OFTP corresponds with the assumptions of socioemotional selectivity theory: When time is perceived as limited people select more emotionally meaningful goals and partners (Fredrickson and Carstensen, 1990; Fung et al., 1999; Carstensen, 2006). In the context of teamwork, workers might have preferred the familiar team in the limited OFTP condition because the situation envisioned an approaching end, triggering negative affect and feelings of uncertainty, (job) insecurity (Gottschalk and Moffitt, 1999), or the upcoming separation from their colleagues. Therefore, spending time with emotionally close teams and team members could help to regulate affect and optimize resource usage by, for instance, coping with the approaching end through social support (e.g., Cohen and Wills, 1985), instead of joining unknown teams with new potential challenges. On a familiar team, workers probably know in advance what they can accomplish, and can adjust their individual effort accordingly. In line with the socioemotional selectivity theory (Carstensen et al., 2003), workers’ benefit in preferring the familiar team might be the contact itself that gives rise to positive feelings through a sense of closeness in a situation of uncertainty (i.e., emotion-oriented goals), whereas joining a new team might be related to potential long-term payoff such as learning or career opportunities (i.e., future-oriented goals; see Fung and Carstensen, 2006). In our experimental situation of an organizational bankruptcy, a long-term payoff was unlikely and the preference for the familiar team increased.

The experimentally extended OFTP condition had almost no effect in comparison to the baseline condition. However, we manipulated temporary OFTP in two different domains that both referred to changes in remaining time at work. The limited OFTP condition was realized using an experimental variation of the organizational future time perspective, whereas the extended OFTP condition referred to the occupational future time perspective. Our variation of the limited OFTP condition requires an imminent reaction of the worker, whereas changes in the extended OFTP condition do not demand a direct action. The chosen scenario of extending temporary OFTP probably was too artificial to affect participants’ team-related preference or simply not relevant for the younger workers. Specifically, participants were invited to imagine a change of legislation enabling a 10-year delay before retirement. These additional 10 years are hard to imagine and seem to be far in the future, and the perceived time is comparable to an open-ended situation, while the limitation of time – represented in our study by bankruptcy and being unemployed within 1 year – is directly noticeable in the present life and affects the workers immediately. Nevertheless, all three experimental contexts affected participants’ team-related preferences as reflected by the order effect of the two randomized scenario conditions. The experimental order “baseline – expansion – limitation” led to no significant increase in the preference for the new team after the baseline condition. However, in the experimental order “baseline – limitation – expansion,” as many participants as in the baseline condition preferred the new over the familiar team again in the extended future time condition (even after preferring significantly more often the familiar team in the limited future time condition). At the same time, the overall preference of the new over the familiar team in the extended future time condition was almost the same as in the baseline condition.

In summary, the contextual limitation of temporary OFTP occurred in addition to the more stable effects of general OFTP. This finding suggests that preferences for familiar over new teams might be influenced by changing the context-related time perspective of workers. Moreover, it suggests that workers’ OFTP might differ intra-individually. For instance, the occupational future time perspective – which comprises the entire working life – considerably differs from the perceived future time perspective on a specific team in a specific company (i.e., organizational teamwork future time perspective; see also, e.g., Stouthard and Peetsma, 1999).

### Practical Implications

This research has various implications for organizations. First, the results show that not just workers’ age but also individual OFTP can affect participants’ team-related preference. In the current study, age effects on participants’ preference for familiar over new teams could only be observed after considering OFTP as a mediating variable. Whether workers can choose a team or are allocated to an existing team, they have their preferences and these preferences are crucial for team members’ motivation and commitment. Awareness of age and time-related preferences for teams might help to better understand workers’ differences in motivation and commitment as members of occupational teams.

In addition to OFTPs specific to certain occupations or business sectors, individual factors such as pregnancy or illness cannot be foreseen. Paying attention to workers’ temporary OFTP helps to anticipate workers’ preference for familiar over new teams. In a rapidly changing business environment it is sometimes advantageous when workers want to work in new teams with changing partners. However, at other times, it is advantageous to work in familiar teams. For instance, in the case of an urgent deadline, the decision to work with known co-workers can save time and money, increase efficiency, reduce misunderstandings, and support the regulation of emotions. As our research shows, workers’ preferences for familiar over new teams could be influenced by the contextual limitation of temporary OFTP. Finally, the knowledge of OFTP variability could improve HR management and provide new avenues for compensating age-related preferences in the context of occupational teamwork.

### Limitations and Future Research

Our study has several limitations. First, the study relies of self-reports. To reduce the influence of common method bias, we followed recommendations by Podsakoff et al. (2012). We assured participants that their answers were anonymous and encouraged them to answer honestly. We confirmed this by underlining that the validity of their personal feedback at the end of the survey depended on their honesty. Furthermore, we collected data from three scenarios in which preferences for familiar over new teams were indicated, and randomized two of these scenarios. This should have minimized the possibility of a common method bias. Second, part of our data is correlational and causal conclusions are therefore limited. However, by using an experimental design with three conditions, we found that the participants’ preference for familiar over new teams changed by contextually limiting the temporary OFTP. Thus, we partly showed the causal influence of OFTP. Moreover, most of the results are in line with our assumed model which extends the theoretical lifespan theories to the teamwork context. Furthermore, the model is based on different empirical studies of the age, lifespan, and general work context (Fredrickson and Carstensen, 1990; Fung et al., 1999; Zacher and Frese, 2011; Hertel et al., 2013; Zacher, 2013). By controlling for health and condition order, we reduced the likelihood of third-variable influences. Third, the order of the experimental conditions correlated with participants’ preference for familiar over new teams in the limited OFTP condition (*r* = -0.11, *p <* 0.05). This means that the workers in the condition order “baseline – expansion – limitation” showed a stronger preference for the familiar team in the limited OFTP condition as compared to the workers in the condition order “baseline – limitation – expansion.” This correlation might suggest that the (experimental) order effect could explain the influence of the limited OFTP condition on participants’ preference for familiar over new teams. To clarify this question, we tested the effects separately for the two condition orders. The experimental influence of the limited OFTP condition appeared in both analyses.^9^ In sum, order of conditions strengthened participants’ preference for the familiar team in the limited OFTP condition but could not completely explain it. Additionally, by using multilevel analysis it was further possible to assess the influence of condition order and OFTP on the between person level and the influence of the contextual extension and limitation of temporary OFTP on the within person level. These analyses also support the effect of the OFTP-limiting manipulation. It should also be noted that the correlation between general OFTP and participants’ team-related preferences was slightly lower in the limited OFTP condition (*r* = -0.12, *p <* 0.05) compared to the baseline (*r* = -0.15, *p <* 0.01) and the extended OFTP condition (*r* = -0.17, *p <* 0.01). However, the differences were not significant. Together, these results support the effect of the experimental limitation of temporary OFTP on the participants’ team-related preference even by considering age, OFTP, and condition order.

This study provides a variety of new avenues for future research. First, similar to research showing that future time perspective is domain specific (i.e., different future time perspectives for life domains such as social, career, and leisure; e.g., Peetsma and van der Veen, 2011; Husman et al., 2016), we assume that time perspective also varies for different domains within the context of occupational work. For instance, different domains of OFTPs could be the tenure of a profession, working for the same company, or the time someone is spending with a working team. Future research might differentiate between general and specific domains on the intra-interindividual level in the work context but also inter-individually between subjects (e.g., Peetsma and van der Veen, 2011; Husman et al., 2016). Further, the interaction and influences of these time perspectives on different outcomes of (team)work, such as effort or performance, might be analyzed. As well as the role of OFTP’s facet of Remaining Time might be considered in the context of age-related differences regarding workers’ preference for familiar over new teams. Second, further mediating and moderating variables which influence age and OFTP might be examined. For instance, Zacher and Frese (2009) analyzed the effects of job complexity and control. For teamwork we assume that autonomy, learning opportunities, and trust of the team members could weaken the relationship between age and OFTP which, in turn, might increase the preference for new teams. Moreover, workers are often free to join the team of their choice. However, at other times the supervisor prescribes the team a worker must join. An interesting question is what happens if a worker has to work in a non-preferred team. Third, contextual factors might be focused on. In this study, we found that the mere imagination of a contextually limited job perspective was sufficient to change participants’ preference for familiar teams. Future work might explore conditions that can also contextually extend temporary OFTP, for example, a scenario in which workers expect an extension of their current work contract (organizational future time) or continued work as freelancers after retirement (occupational future time).

## Conclusion

Based on socioemotional selectivity theory, we tested the relationship between age mediated by OFTP and participants’ preference for familiar over new teams. In addition, we experimentally examined the contextual variability of temporary OFTP and its effect on participants’ team-related preferences. Overall, our findings show that in the work context the relationship between age and participants’ preference for familiar over new teams can be shown only by considering OFTP as mediator. Furthermore, the results establish new ways to affect workers’ team-related preference by influencing context-based OFTP. Moreover, it shows the causal influence of OFTP on participants’ preference for familiar over new teams in a limited OFTP condition.

## Author Contributions

LG: Substantial contributions to the conception or design of the work, to the acquisition, analysis, and interpretation of the data for the work; drafting the work; final approval of the version to be published; agreement to be accountable for all aspects of the work in ensuring that questions related to the accuracy or integrity of any part of the work are appropriately investigated and resolved. GH: Substantial contributions to the conception or design of the work, and to interpretation of data for the work; revising it critically for important intellectual content; final approval of the version to be published; agreement to be accountable for all aspects of the work in ensuring that questions related to the accuracy or integrity of any part of the work are appropriately investigated and resolved.

## Conflict of Interest Statement

The authors declare that the research was conducted in the absence of any commercial or financial relationships that could be construed as a potential conflict of interest.

## Footnotes

1 https://psyweb.uni-muenster.de

2 Initially, 6,124 panel members met the criteria of being between 18 and 67 years old and working, and were thus invited to participate. Non-respondents did not differ demographically from the respondents.

3 Indeed, the measured OFTP scores were not different as a consequence of the experimental manipulation using imagined extended or limited OFTPs, M_order(baseline-limitation-expansion)_ = 3.94 and M_order(baseline-expansion-limitation)_ = 3.97.

4 This study is available on request from the first author.

5 A full list of variables collected in this study is available on request from the first author.

6 https://psyweb.uni-muenster.de

7 Comparing the correlations of OFTP with participants’ preferences for familiar over new teams in the baseline and in the limited OFTP condition showed non-significant differences, *z*(*N* = 454) = 0.84, *p* = 0.20; and similar results were found comparing the correlations of OFTP with participants’ preferences for familiar over new teams in the extended and in the limited OFTP condition, *z*(*N* = 454) = 0.50, *p* = 0.31.

8 The effect between age and Focus on Opportunities (path a), *b* = -0.06, *t* = -9.97, *p <* 0.001, and Focus on Opportunities and participants’ preference for familiar over new teams (path b), *b* = -0.30, *Z* = -4.17, *p <* 0.001, were significant. However, the direct effect of age on preference for familiar over new teams after controlling for the mediator Focus on Opportunities (direct path c’) was non-significant, *b* = -0.02, *Z* = -1.55, *p* = 0.12. The effect between age and Focus on Limitation (path a), *b* = -0.09, *t* = -13.77, *p <* 0.001, was significant. However, all other paths were non-significant; Focus on Limitation and participants’ preference for familiar over new teams (path b), *b* = 0.02, *Z* = 0.29, *p* = 0.77; age and participants’ preference for familiar over new teams after controlling for the mediator Focus on Limitation (direct path c’), *b* = 0.01, *Z* = 0.44, *p* = 0.66.

9 The multilevel analyses showed that for both condition orders the contextual expansion of temporary OFTP had no influence on participants’ preference for familiar over new teams. The results are for the condition order 0 (baseline, expansion, limitation; n_order0_ = 237) *b* = -0.05 *p* = 0.83, OR = 1.05; for the condition order 1 (baseline, limitation, expansion; n_order1_ = 205) *b* = 0.34, *p* = 0.18, OR = 0.71. However, the contextual limitation of temporary OFTP led for both condition orders to a higher preference for the familiar over the new team, condition order 0 (baseline, expansion, limitation; n_order0_ = 237) *b* = 0.65, *p <* 0.001, OR = 0.42; for the condition order 1 (baseline, limitation, expansion; n_order1_ = 205) *b* = 1.05, *p <* 0.001, OR = 0.35, even when controlling for age and general OFTP.
